# Supplementary material for: Real-time 3D tracking of swimming microbes using digital holographic microscopy and deep learning
Source: PLoS One. 2024 Apr 26;19(4):e0301182. doi: 10.1371/journal.pone.0301182 (PMC11051601; doi:10.1371/journal.pone.0301182)
Supplement: Attachment — Submitted filename: Response letter_LW_SM_LW.pdf [file pone.0301182.s001.pdf]

Dear Dr. Nadeau,

Thank you for your invitation to resubmit a revised manuscript, the reviewer comments were insightful and helped us improve the manuscript. We outline our responses to the reviewer feedback below.

First we thank ‘reviewer one’ for their feedback. We hope we have addressed their points, which we deal with in order:

1. An exploration of the method's robustness across different cell morphologies within the same species. It's crucial to ascertain if cells with varied morphologies yield consistent results and how the technique fares with cells oriented in different planes.

We have highlighted that the method used to gather the training data is robust to changes in cell morphology and orientation in the plane. Indeed, the setup with this particular magnification is designed so that cells appear as point-like scatterers, regardless of the inevitable minor differences in shape/size between individuals (though this information will naturally be included in our training data). The training data does not discriminate between individuals on this basis, so the machine learning approach that is trained on the data will not be able to do so either. We agree that the use of individual cell morphology could (in principle) give more information about phenotypic variability within a population, but this lies outside the scope of our study, which is aimed at capturing motility. We have discussed these and other aspects of the classification problem in lines 350-367 of the revised manuscript.

2. An assessment of the technique's efficacy with denser samples.

We thank the reviewer for their suggestion; we have included some analysis of the technique applied to denser samples in Figure 4, Table 1 and paragraphs on lines 255 – 272.

3. A detailed discussion on the specificity and robustness of the method concerning the system's variables, such as the modulation transfer function of the objective lens, the coherence properties of the light, and the camera's noise characteristics.

We have discussed this topic in greater detail in the text leading up to the conclusions section. Strictly speaking our method, in common with other visual AI, is reliant on the particular setup, and the model would have to be trained on data particular to that setup for it to work. Fortunately, this is unlikely to extend to the identity of specific components: we could swap the objective lens in our microscope with an equivalent part from a different system without compromising the method. Moreover, adaptation of our method to a new optical system should be much faster and more efficient than our initial setup, using the principles of transfer learning based on our trained model, as discussed in lines 316-333.

4. Is this approach 10 times faster than the conventional method when talking about a larger order? If so, does this include the time of the reconstruction process with the other method?

Yes, assuming by larger order the reviewer is referring to more objects per frame (i.e. denser samples), there is no increase in time per image for the yolov5 model detecting more objects. We have clarified this on line 272-273.

5. A schematic representation of the method, including the preparation and training phases, would be highly beneficial for comprehension.

We thank the reviewer for their suggestion; Fig. 1 has been expanded to include a brief overview of the sample preparation and algorithm training process.

6. In Fig 4c, it's unclear whether these are selected tracks. Additionally, the maximum value for mAP appears to end at 0.5; further explanation and an analysis of Fig 3's weaknesses would enhance understanding.

We have included a note in the caption to the relevant figure explaining that these are selected tracks. An explanation of mAP and other metrics has been added to the figure caption of Figure 3.

7. Suggestions for optimizing the training to attain higher mAP values would be valuable.

We thank the reviewer for this suggestion, and have suggested ways of improving the model and metrics in line 235-243.

8. The article also needs clarification regarding the number of cells per image as mentioned in lines 104 and 121.

We have added clarification of the number of cells in each frame in these lines (now 108, 121)

9. Additionally, there are several typographical errors that need rectification

We thank the reviewer for drawing our attention to these presentational issues, and have attempted to remove remaining typographical errors.

Reviewer two's comments were also most welcome and helped us refine our paper:

10. Major 1, Fig 2, L155: The figure, its caption, and the text do not clearly communicate why and how the systematic vertical offset was applied. The figure seems to represent an incorrect straight-line fit to the data shown at first glance. An improvement would be to include both pre- and post-offset fits. The text should describe how the vertical offset was calculated; the slope and intercept of the fit itself could also be reported, considering its simplicity. It is also unclear whether this offset is strictly necessary, since the 4th-order polynomial correction applied to the model post hoc could also compensate for this error (unless the argument is that the model itself is simply worse without the offset). However, since re-training the deep learning model requires significant effort, a clearer explanation here is sufficient.

We thank the reviewer for their comments here; this is one of the more nuanced areas of our calibration and so we have revisited the description in an attempt to increase the clarity of exposition. We have included both pre and post vertical offset in Fig 2 and have added line 175-178 to explain that without the offset the bounding box sizes for cells at axial positions under ~25um would be negative.

11. Major 2, L79-82: While this particular instrument's configuration allows the microbes to be treated as point-like scatterers, a discussion of the method's limitations with high-magnification instruments where the size of the microbe itself contributes to the bounding box size is critical (e.g. a large microbe at the focal plane could have the same bounding box size as a small microbe at some z). This would be especially challenging in an environment with a diverse population of microbes with different morphologies, and the problem is certainly salient for 'agnostic' biodetection problems where the scale of the objects of interest is not known *a priori*. In these cases, we would most likely have to combine object detection with some sort of localised spatial frequency information to corroborate the objects' distance from the focal plane.

(see also 1. above). However, we re-emphasise that our work is limited to a configuration that allows the microbes to be treated as point-like scatterers; this should still allow detection of motile objects (regardless of the magnification) and would allow a quantified discrimination between swimming and Brownian motion. (lines 366-368).

12. Major 3, L204: The results section of this manuscript does not report any quantitative metrics for tracking performance. While the manuscript may not include other methods to compare to, a quantitative performance metric is important for objective evaluation and for future works seeking to improve upon these results on the released data set. An advanced option would be the alpha and beta metrics used for the Particle Tracking Challenge (Chenouard, et al., 2014, Nature Methods), but a simple RMSE would also be sufficient. Additionally, performance metrics on the training, validation, and test sets would also indicate or dispel overfitting.

We thank the reviewer for their suggestion, and apologise for this oversight. We have added RMSE for tracks pre and post 4th order correction (line 293-295), added performance metrics for all training sets and dense data (Table 1), and directed the reader to the reference suggested for cases where a more detailed investigation may be sought.

13. Major 4, L217: The 4th-order polynomial correction (which could also be described as a form of model calibration) should be fit on the results from the training data, then applied unchanged to the results from the unseen data. By fitting the correction directly on the unseen data, it hides the possibility that this bias/deviation introduced by the model cannot be corrected in a generalizable fashion (as suggested in L229). This is a critical correction that should require little effort.

We thank the reviewer for the suggestion. The 4th order calibration has been fit to validation data and then applied to test data, as shown in what is now Fig 5(a) and (b).

Minor points

We thank the reviewer for their attention to these presentational aspects of our manuscript and we believe that the typographical errors identified have been addressed at their relevant locations. We also thank reviewer two for their suggestion of additional references, which we have incorporated. We have also added some additional references that we believe help elucidate the process where necessary and motivate the work. These are highlighted in the revised manuscript.
